# Does Bisphenol A (BPA) Exposure Cause Human Diseases?

**DOI:** 10.3390/biomedicines12122678

**Published:** 2024-11-25

**Authors:** T. Peter Stein

**Affiliations:** Rowan-Virtua School of Translational Biomedical Engineering and Sciences and School of Osteopathic Medicine, 2 Medical Center Drive, Stratford, NJ 08084, USA; tpstein@rowan.edu

**Keywords:** autism, ADHD, PCOS, Parkinson’s, Alzheimer’s, bisphenol A, BPA, glucuronidation, DEHP (diethyl hexyl phthalate)

## Abstract

Background: Autism spectrum disorders (ASDs), attention-deficit disorder (ADHD), Parkinson’s disease (PD), polycystic ovary disease (PCOS), and Alzheimer’s disease (AD) have all been linked to exposure to bisphenol A (BPA). Methods: This paper is a review and discussion of the published literature. Results: Animal studies have shown BPA to be a broad-spectrum endocrine disruptor. BPA is metabolized via the glucuronidation pathway, which involves the addition of glucose to the target molecule, and is catalyzed by uridine 5′-diphospho-glucuronosyltransferases (UGTs). Evidence of compromised glucuronidation has been found for ASD, DHD, PD, and PCOS. Genetic polymorphisms that alter the catalytic activity of the UGTs and efflux transporters involved are common. There are two ways to interpret the findings of associations between BPA glucuronidation efficiency and disease, a ‘direct’ pathway and an ‘indirect’ pathway. With the ‘direct’ pathway, free BPA is the actual causative agent. Compromised BPA detoxification leads to higher concentrations of free BPA in vulnerable tissues. Decreased BPA detoxification leads to increased exposure of vulnerable tissues to free BPA, where it can function as an endocrine disruptor. With the ‘indirect’ pathway, BPA is not the causative agent. BPA serves as a marker for the decreased glucuronidation efficiency of another unknown compound of endogenous origin detoxified by a similar combination of UGTs and efflux transporters as BPA. It is this compound(s), acting as an endocrine disruptor, that leads to a metabolic environment that favors disease development over an extended time period. Conclusion: A review of the existing literature supports the indirect ‘marker’ hypothesis over the ‘direct’ hypothesis.

## 1. Introduction

The objective of this article is to review the relationship between bisphenol A (BPA) and five common human diseases: autism spectrum disorders (ASDs), attention-deficit disorder (ADHD), Parkinson´s disease (PD), polycystic ovary disease (PCOS), and Alzheimer’s disease (AD). All have long incubation periods, are not acutely lethal, and have been shown to be associated with exposure to BPA. Multiple authors have proposed that there are commonalities between neuro-developmental (ASD and ADHD) and neurodegenerative diseases (PD and AD) [1,2,3,4,5,6,7,8,9,10,11]. An association with BPA is one of these commonalities.

This article reviews (i) the prevalence of the five diseases, (ii) the relationship between the diseases and BPA, (iii) how human exposure can occur, (iv) BPA metabolism, and (v) the mechanisms by which BPA can relate to human disease. The emphasis is on human data because there are no generally accepted animal models for the five diseases.

## 2. Disease Prevalence

During the last century, there has been a substantial increase in the observed incidence of some diseases which previously were rare. The examples discussed in this paper are autism spectrum disorders (ASDs), attention-deficit disorder (ADHD), Parkinson´s disease, polycystic ovary disease (PCOS), and Alzheimer’s disease (AD). The common feature is that all have been associated with exposure to bisphenol A (BPA).

Placing an exact number on just how large the increases have been is problematic. A century ago, most people did not live long enough for AD and PD to be a major factor in overall mortality. The childhood mortality rate was very high. Childhood diseases such as ASD and ADHD were regarded as minor behavioral aberrations for most children, considering the numerous life-threatening diseases then prevalent. ‘Discipline’ was often the treatment of choice for ‘behavioral’ problems. For PCOS, a standardized definition was not agreed on until the end of the 20th century.

Adding to the complexity of integrating past frequency data with present data are shifting disease definitions, along with increased access to an ever-improving medical system and increased disease detection capabilities. With better living conditions and diets, there have been dramatic reductions in mortality for major diseases such as malnutrition, cholera, plague, etc., allowing other non-life-threatening diseases to become more apparent. As lifespan has increased, the frequency of degenerative diseases has increased. The net result is that reliable data are only available for the last ~20 to 30 years. During that time, worldwide BPA production has increased by ~5–7%/yr [12,13,14].

In that time period, the Autism and Developmental Disabilities Monitoring Network estimated that the prevalence of ASD in children aged 8 years increased from 1 in 150 in 2000 to 1 in 36 by 2020 [15]. Similar data from the CDC reported ADHD to have increased between 1997 and 2016 from 6.1% in 1998 to 10.2% and to 11.3% by 2022 [16,17]. A meta-analysis by Zhu estimated the worldwide incidence of Parkinson’s disease to be 1.5 per 10,000 in 1996, 2.8 per 10,000 in 2012, and 4.7 per 10,000 in 2020 [18]. Alzheimer’s disease was first described by Alzheimer in 1907 [19]. A century later, the CDC reported the incidence in the USA per 100,000 to be 18.1 in 2000 and 30.5 by 2018 [20]. Past prevalence information on PCOS has been difficult to obtain because PCOS, at some level, was always present, but the diagnostic tools were not available [21]. The worldwide incidence rate has been estimated as 46 per 100,000 in 1990 and 70 per 100,000 in 2019 [21]. In the UK, the incidence of PCOS per 100,000 patient-years increased from 122 in 1990 to 220 in 2019 [22]. These intra-disease values are not strictly comparable because the reference populations vary from the same gender and age cohort to the population at large. A better estimate of the increases can be obtained by comparing the recent annual rates of increase using data where all measurements were made by a single team (Table 1).

## 3. Association of Diseases with BPA

A substantial increase in the amount of man-made chemicals introduced into the environment has coincided with the increase in these diseases. Is there a connection? All of these diseases unquestionably have multiple etiologies. The questions being asked in this review are as follows: does one of those etiologies include an environmental component, and, if so, is BPA a possibility, and how significant is its role?

Etiologies involving exposure to xenobiotics are attractive because it is generally accepted that chemicals introduced into the environment by human activity can have adverse effects on human health [23,24,25,26]. There is a vast amount of literature on animal data supporting the role of xenobiotics of human origin in the etiology of human diseases [1,27]. For some of these diseases, definite linkages have been found. Some examples are lead [28], mercury [29], pesticide poisoning [30], and tobacco smoke [31]. For other diseases, such as the diseases considered here, links are strongly suspected but not proven.

### BPA as a Toxic Xenobiotic

BPA is a lipophilic and relatively inert aromatic compound of moderate molecular weight. The major industrial use of BPA is in the manufacture of polycarbonate plastics and epoxy resins [25,32,33,34]. Epoxy resins are used to coat the inside of many food and beverage cans. Polycarbonate plastics have multiple uses because they are versatile, malleable, and cheap to make. Some minor uses of BPA are in polyvinyl chloride (PVC) manufacture as an antioxidant and plasticizer [12,13,25,34]. In spite of concerns about its safety, manufacture, and usage worldwide, BPA production continues to increase at a rate of about 5%/year [12,13,14]. The principal routes of exposure are believed to be dietary through the ingestion of food products via contaminated packaging, although there is some evidence that inhalation and personal products could also be important [26,35,36].

BPA is released into the environment when plastics derived from BPA degrade [37]. Additionally, BPA can leech out when it is not part of a polymer structure but is present in the role of a plasticizer and/or antioxidant. Another potential source is the contamination of plastics with free precursor BPA. This BPA leaches out easily and is more likely to be a problem with poor-quality polycarbonates [38]. These processes are the principal sources of BPA in the environment, drinking water, and food chain [39]. Thus, exposure and uptake via ingestion, inhalation, and dermal contact are continuous.

BPA is a major industrial chemical with an annual value estimated to be USD 20 billion [12,13,14]. Whether BPA is or is not toxic at current levels of exposure is controversial [40,41,42]. Some years ago, Vom Saal made the interesting observation that most of the published studies demonstrating toxicity came from academia, governments, and non-profits. Studies sponsored by the plastics industry were prone to report BPA as being safe at current usage levels. Some of the discrepancies between academia and industry may be due to the wide range of BPA concentrations tested as well as to different experimental models [43,44,45]. Other complicating factors in relation to animal studies on the human situation are non-oral routes of administration in many experiments, different doses, the absence of dose–response relationships, and small numbers of test animals [43,44,45]. It is also problematic that the animal data show that under the appropriate conditions, BPA interacts with many molecular processes [1,27,46,47,48]. Government-run public health agencies charged with assessing BPA’s safety took the middle road, involving regulation with some restrictions.

In vivo human metabolic studies are very limited. Most exposure ‘risk data’ come from epidemiological studies on large populations that identified multiple associations but failed to rank them in order of relative importance. Many possible mechanisms have been identified in animal studies. Since none of the animal models have been shown to accurately reproduce the human disease situation, the identification of the actual responsible pathway is problematic. A recent review of the problem by Rebelledo-Solleiro summarized the situation as involving multiple diseases with numerous possible mechanisms, each with support from animal studies (Table 2; [49]).

Human diseases are caused by chronic very low-level exposure and can take years to become observable. Chronic low-level exposure leading to minor aberrations in a biochemical pathway is difficult to detect. It is only within the last 50 years that it has been possible to measure free BPA in the environment and tissues. Most scientists cannot afford to wait for long-term outcomes, which low-level exposure studies require. The system is set up to produce rapid results, and this requires high-dosage experiments. (Consume enough of any nominally harmless substance, and it will cause problems. Excess food is an example). The relevance of high-dose studies has been queried [41,43,50].

## 4. BPA Metabolism

The purpose of xenobiotic metabolism is to enable the rapid excretion of potentially toxic compounds from the body, be they of exogenous or endogenous origin. In most instances, this involves increasing water solubility to enable excretion in urine. Xenobiotic metabolism is commonly divided into two phases. In phase 1, enzymes such as cytochrome P450 oxidases add reactive or polar groups to the target molecule. In phase 2, reactions involve adding a glutathione, sulfate, or glucose group to the products of phase 1 to further increase their water solubility.

In order to exert toxicity, a chemical has to enter into a cell. Chemicals penetrate into and out of cells either by unregulated passive transport or by active transport processes. Active transport is a tightly regulated process and a key determinant in the disposition and metabolism of xenobiotics [51]. There are two major families of transporters. The solute carrier (SLC) transporter family functions mainly as uptake transporters, and ATP-binding cassette (ABC) transporters are primarily efflux transporters. Several members of both families are involved in xenobiotic uptake and product removal from the cells and so can affect the concentrations of original and derived products in the circulation [52,53]. This process is particularly important in modulating drug potency. Examples of efflux transporters modulating drug potency include the breast cancer resistance protein (BCRP; ABCG2), P-glycoprotein (P-gp; ABCBl), multidrug and toxin extrusion proteins (MATEs), and multidrug resistance-associated proteins (MRPs; [54]).

Phase 2 reactions are catalyzed by three sets of transferase enzyme systems: glutathione-S-transferases for glutathione, sulfotransferases for sulfation, and uridine 5′-diphospho-glucuronosyltransferases (UGTs) for glucose. About 90% of BPA intake is detoxified by the glucuronidation pathway and most of the remainder as sulfate [55,56]. Two other essential steps are associated with these transformations: the uptake of the xenobiotic into the cells and the export of the final water-soluble product from the cells [38,54,57].

BPA that is not converted into glucuronide (or sulfate) can penetrate into vulnerable cells. The BPA can bind to multiple sites, especially estrogenic receptors (ERα and -β), nuclear receptors, GPR30, androgen receptors, thyroid hormone receptors (TRα and -β), estrogen-related receptor gamma (ERRγ), and glucocorticoid receptors [58,59,60,61]. There are also animal data showing that BPA can have anti-androgenic effects [46,62,63,64]. The breadth of the potential mechanisms in Table 2 and the above listing of potential BPA-binding sites is significant. It illustrates the complexity of identifying exactly which one(s) could be responsible for a given disease. Any of these receptors could contribute to the adverse effect(s) of BPA on humans. A discussion of the possibilities is beyond the scope of this review. For a recent detailed review, see the review by Cimmino et al. [46].

The paragraphs that follow present details of the glucuronidation pathway that are relevant to BPA glucuronidation. The pathway involves three steps: (1) the uptake of BPA into the cell where detoxification occurs; (2) the glucuronidation of the target molecule by UDP-glucuronosyltransferases (UGTs) into hydrophilic glucuronides [54,57,65,66,67,68]; and (3) the transport of glucuronide out of the cell by efflux transporters [54,57,68,69].

### 4.1. Step 1: Uptake of BPA into Cells

The liver is the major, but not the only, site for the biotransformation and excretion of xenobiotics. Once in the body, BPA is rapidly taken up by the liver with a high first-pass efficiency and processed [25,70,71,72]. Other tissues have the ability to detoxify BPA, but their quantitative role is minor. The uptake of circulating compounds from the blood can either be by active transport or passive diffusion. Active transport involves specific transport enzymes, which are located on the basolateral membranes of hepatocytes by OATPlBl (SLCOlBl), OATP1B3 (SLCO1B3), and OATP2Bl isoenzymes [73]. BPA is a neutral, lipid-soluble molecule, so passive uptake is also likely [46,72,74].

Hepatic uptake, with possibly some contribution from the gut, is not completely efficient. If it were, BPA would not be present in the circulating blood or other tissues, including the brain [30,46,47]. How much BPA penetrates into brain cells depends on the interplay between multiple factors, including intake, hepatic glucuronidation efficiency, and the efficiency of uptake by other tissues. BPA can penetrate into brain tissues via active transport and, because BPA is lipophilic, also by passive diffusion. Because BPA can penetrate into cells via diffusion as well as active transport, compromised uptake is unlikely to be a rate-controlling factor in BPA metabolism [46,72,74].

Either decreased efficiency of glucuronidation or glucuronide excretion could result in longer residence times of bioactive moieties in a tissue. This can be good (increased drug potency) or bad (increased toxicity). UGTs control glucuronide production, while efflux transporters control the rate of glucuronide removal from the cells. The two steps act in concert with each other to give the measured rate of glucuronidation [54,57,75].

### 4.2. Step 2. Glucuronidation by UGTs

Endogenous UGT substrates include bilirubin, bile acids, lipid acids, steroids, thyroid hormones, and lipid-soluble vitamins [76,77,78]. Exogenous substrates include most xenobiotics, such as miscellaneous plant compounds, and many industrial chemicals, such as drugs, food additives, fertilizers, and products associated with the plastics industry.

The superfamily of human UGT proteins comprises 22 functional isoenzymes, which are divided into four gene families, UGT1, UGT2, UGT3, and UGT8, according to the sequence similarity [79,80]. Polymorphisms are common. The various isoenzymes differ in their tissue distributions [67,80,81]. The isoenzyme patterns and distributions also show relationships with both geographic and ethnic backgrounds [67,82]. Pharmacogenetic variability has been found in virtually every UGT family member. The different UGTs have numerous and overlapping substrates [82].

Multiple UGT isoenzymes are able to glucuronidate BPA. The major contributor is from UGT2B15, along with contributions from UGT1A1, UGT1A3, UGT1A9, UGT2B4, and UGT2B7 [75,83,84]. The glucuronidation of BPA by UGT2B7^H268Y^ is less efficient than with the wild enzyme [85,86]. Thus, while the mechanism is common, execution is carried out by a multitude of closely related enzymes, leaving much scope for individual variability and extra processing capacity if needed. Differences in net glucuronidation efficiency could, therefore, be due to either an isoenzyme difference or a difference in the isoenzyme distribution pattern.

Genetic polymorphisms that alter the catalytic activity of UGTs can be clinically important [67,87]. Differences in the efficiency of the glucuronidation processes can affect the substrate concentrations and, consequently, their effects [65]. An increased residence time in the bloodstream and/or greater concentration of a free compound in the bloodstream can increase tissue exposure to the drug/toxinogen and vice versa [65,67,82]. Because of the importance of drug effectiveness and toxicity in clinical medicine, studies on UGT polymorphisms and their effects on glucuronidation efficiency have focused on their relation to drug potency. The rates of drug degradation (or activation) can have a major impact on the effectiveness and safety of a drug [66,67].

An example is irinotecan, a naturally occurring cytotoxic alkaloid widely used in the treatment of non-small cell lung cancer. UGT typing is used clinically to determine the irinotecan dosage. The UGT1A1G71R (G > A; rs4148323) polymorphism is associated with the reduced effectiveness of irinotecan-based regimens in non-small cell lung cancer and is a predictor for severe adverse events. The UGT1A1∗6 and UGT1A1∗28 polymorphisms reduce the activity of UGT1A1 by as much as 40% to 60% [88].

### 4.3. Step 3: Efflux Transporters

Glucuronides are exported from their source cells by efflux transporters. The role of efflux transporters is the prevention of the cellular accumulation of potentially toxic substances in the cells by exporting the neutered glucuronide [75,89]. Compromised export decreases glucuronidation because glucuronide backs up in the cells. Although glucuronidation is the more studied of the two processes, the net excretion of the product glucuronide requires the cooperation of both processes: conjugation and excretion [54,57,68,69]. If either step is compromised, the net result is the same: decreased glucuronidation.

The transportation of glucuronides out of cells is an active transport process and involves ATP hydrolysis by the transporter [90]. The transporters are members of the ABC protein family [69]. The ATP-binding cassette (ABC) superfamily is composed of multiple ATP-hydrolyzing enzymes that actively transport a broad range of substrates out of cells [75,91]. Among the transporters encoded by the multidrug resistance gene 1 (MDR1) are multidrug resistance-associated proteins (MRPs) (MRP1, MRP2, MRP3, and MRP4), the breast cancer resistance protein (BCRP), and the hepatic bile salt export pump (BSEP; [38,92]).

MRPs are the major efflux transporter family for phase II metabolites [93,94]. Although several ABC transporters, including MRP2, MRP3, and BCRP, can transport BPA, MRP3 is the dominant transporter of BPA [38,69]. MRP3 has a high affinity for glucuronides [38,95,96]. Genetic alterations in the MRP3 gene occur, and they can affect MRP3′s enzymatic activity [57,97].

An example of how genetic variations in efflux transporter genes can affect transporter function is the role of MRP3 isoenzymes in bile production [98]. Droge et al. sequenced MDR3 genes for 215 patients with cholestatic liver diseases [98]. The distribution of four common SNPs, MRP3^L59L^, MRP3^N168N^, MRP3^I237I^, and MRP3^R652G^, in patients with cholestasis was compared with a reference population. They found a highly significant association of MRP3 mutations and the common single-nucleotide polymorphism (SNP) p.I237I with elevated liver enzymes, gallstones and intrahepatic cholestasis, liver cirrhosis, and hepatobiliary malignancies [98].

Although the conjugation step is believed to be the more important one and is the more studied process, detoxification requires the cooperation of both processes, either of which could be rate-controlling [54,68,69]. Thus, the elimination of a xenobiotic via glucuronidation is controlled by two processes: the formation of glucuronides by UGT enzymes and then the excretion of these glucuronides by efflux transporters located on the cell surfaces [38,69,75,99]. Polymorphisms in transport proteins are common with multiple physiological effects, including disease and pharmacotherapy [100].

## 5. BPA Glucuronidation and Human Diseases

To date, there has been no complete study directly linking actual measured decreases in BPA glucuronidation efficiency with isoenzyme differences in either the glucuronidation step or the efflux step with actual disease. What has been carried out is measuring either the relationship between isoenzyme distributions and disease and then inferring differences in enzyme activity somewhere in the overall glucuronidation pathway or measuring the relationship between disease and glucuronidation efficiency and inferring that any differences found were due to isoenzyme distribution differences. There are four published (partial) examples, each dealing with a major human disease. The four studies are discussed below.

### 5.1. Study #1: Polycystic Ovary Disease (PCOS)

PCOS is known to be associated with BPA [101,102,103,104]. A PCOS study by Luo et al. linked PCOS and BPA with UGT isoenzyme distribution pattern differences. They genotyped 357 Chinese women (119 PCOS cases and 238 controls) for UGT1A1G71R, UGT2B7^H268Y^, and UGT2B1^5D85Y^ polymorphisms [105]. A complex but different pattern from the controls was found. A higher proportion of the TT genotype of UGT2B7^H268Y^ was associated with an increased risk of PCOS, whereas the TT genotype of UGT2B15 D85Y was associated with a decreased risk of PCOS. The UGT2B7^H268Y^ SNP was associated with an increased risk of PCOS and with the plasma BPA concentration.

The results were consistent with epidemiological data showing an association between BPA and PCOS and supported the role of genetic differences in modulating BPA glucuronidation efficiency. However, because they did not actually measure the glucuronidation efficiency, a direct linkage between BPA glucuronidation efficiency and PCOS could only be inferred. Differences in glucuronidation efficiency could be due to isoenzyme distribution patterns rather than a ‘different’ isoenzyme. This seems to be the case for PCOS, where both patients and controls have the same set of isoenzymes, but the distribution differed, and PCOS patients had a higher proportion of the less efficient genotype of UGT2B7^H268Y^. In situations where a single protein is responsible for a reaction, the substitution of another variant can easily be detected. Where multiple variants are involved, unraveling the differences between patients and controls requires the measurement of the relative abundance and activity of each isoenzyme. Like the direct substitution of one genotype for another followed by disease, the distribution pattern of a family of isoenzymes is genetically determined.

### 5.2. Study #2: Parkinson’s Disease (PD)

The characteristic feature of PD is the loss of dopaminergic neurons in the midbrain, together with the intracellular accumulation of a-synucein. Estrogens are known to increase dopamine production, and BPA is an estrogen mimic, so as Rebolledo-Solleiro et al. pointed out, there is a potential mechanism for an association between BPA and PD [49,106].

Landolfi et al. measured the concentration of free and conjugated BPA in the blood of patients with PD [107]. Spouses were used as the controls. Since exposure to BPA could have happened decades earlier, and so could not be documented, the use of spouses was a reasonable solution. This allowed them to assume that spouses shared a common environment for many years and, therefore, could serve as environmental controls.

The key finding was the percentage of BPA conjugated (=glucuronidation efficiency) with PD was 76.7% ± 2.7% versus 83.6% ± 4.4% (*p* < 0.0001) for the controls. They attributed this reduction to ‘the pharmacological burden’ from the varied list of anti-PD drugs to which they were exposed. We suggest an alternate explanation: a genetically determined reduction in glucuronidation efficiency either because the actual glucuronidation step is less efficient due to differences in UGTs or the export of glucuronide out of cells by efflux transporters is less efficient.

### 5.3. Studies #3 and #4: ASD and ADHD

The etiology of autism spectrum (ASD) and attention-deficit/hyperactivity (ADHD) disorders are multifactorial. Numerous epidemiological studies have shown both ASD [23,108,109,110,111,112,113] and ADHD [114,115,116,117,118] to have strong associations with both genetic components and environmental factors, including BPA. Multiple mechanisms have been proposed for the role of BPA; most involve BPA acting as a weak endocrine disruptor [116,119,120,121].

A study by Stein et al. focused on measuring the efficiency of glucuronidation in children with ASD, along with ADHD healthy control children [122]. The glucuronidation efficiencies for BPA were reduced by 11% for ASD (*p* = 0.021) and 17% for ADHD (*p* < 0.001). These numbers are similar to those reported by Landolfi et al. for PD [107].

The analytical methodology used yielded glucuronidation efficiency data for 12 varied compounds. The twelve compounds included five ‘plasticizers’ (BPA and four metabolites of DEHP (diethylhexyl phthalate), i.e., MEHP (methyl ethylhexyl phthalate)—the primary metabolite of MEHP—and three secondary metabolites, mono-(2-ethyl-5-oxohexyl) phthalate (5-oxo MEHP), mono-(2-ethyl-5-carboxypentyl) phthalate (5-CX MEPP), and mono-(2-ethyl-5-hydroxyhexyl) phthalate (5-OH MEHP)); a steroid (cortisol); two bile acids (glycocholic acid (GDA) and glycodeoxycholic acid (GCA)); two vitamin E metabolites (2,5,7,8-tetramethyl-2-(2′-carboxyethyl)-6-hydroxychroman (α-CEHC) and 7,8-trimethyl-2-(beta-carboxyethyl)-6-hydroxychroman (γ- CEHC)); and two plant compounds (the fruit flavonoid naringenin and salicylate). 

Of the 12 glucuronidation efficiencies examined, only BPA showed statistically significant associations with reduced glucuronidation efficiency for both ASD and ADHD. Similar but not significant trends were found with MEHP. No relationships were found with any of the other 10 compounds [122].

Studies on BPA are often conducted in parallel with studies on phthalates [1,27,123,124]. Both BPA and DEHP are medium-sized aromatic molecules widely used by the plastics industry. BPA is mainly used as part of the structure of the plastic, with a small amount used as a plasticizer. DEHP’s primary use is as a plasticizer. Plasticizers are additives used to modulate the physical properties of plastics. As additives, they are not chemically bonded to the plastic and so can leach out.

The most common phthalate is DEHP (diethylhexyl phthalate). The bioactive form of DEHP is the product of the first step in its metabolism, mono ethylhexyl phthalate (MEHP; [125,126]). Like BPA, phthalate detoxification and excretion involve the glucuronidation pathway. The epidemiological findings for DEHP are very similar to those found for BPA, including associations with ASD, ADHD, PCOS, PD, and AD. Compromised MEHP glucuronidation has also been found to be associated with ASD and ADHD [106,123]. The associations found with DEHP do not appear to be as strong as those with BPA. Investigators often elect to study both [1,124,127,128,129].

**Figure 1:**
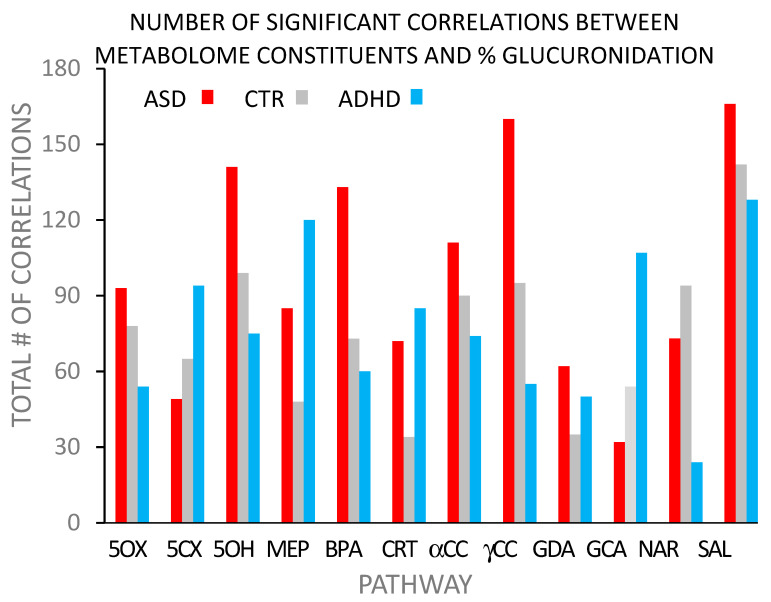
Percentage of total number of Spearman’s rank-order correlations between glucuronidation efficiency, either +v or −ve, by glucuronidation pathway vs. individual metabolome compounds. Abbreviations: 5OX, 5-OXO MEHP; 5CX, 5-CX MEHP; 5OH, 5-OH MEHP; CRT, Cortisol; GDA, Glycocholate; GCA, Glycodeoxycholate; NAR, Naringenin; SAL, Salicylate.

An examination of the fold differences between ASD or ADHD and control children with BPA glucuronidation showed multiple differences across the major metabolic pathways. Figure 1 examines the relationship of the 12 glucuronidation pathways with the metabolome and whether those relationships were impacted by the clinical state. Spearman’s rank-order correlations were calculated for all compounds present in the metabolome for >50% (n = 287) abundance for each of the 12 glucuronidation efficiencies. Only correlations that were significant at the >0.05 level were included in the tabulations and plot (Figure 1; [122]).

Figure 1 shows the total number of statistically significant sign-independent Spearman’s correlations between the 692 metabolome constituents and each of the 12 glucuronidation pathways for the ASD, CTR, and ADHD groups. Similar patterns were found when two other ‘superfamily’ amino acids and xenobiotics were plotted separately. The patterns were varied, complex, and lacking in any obvious specificity.

**Figure 2:**
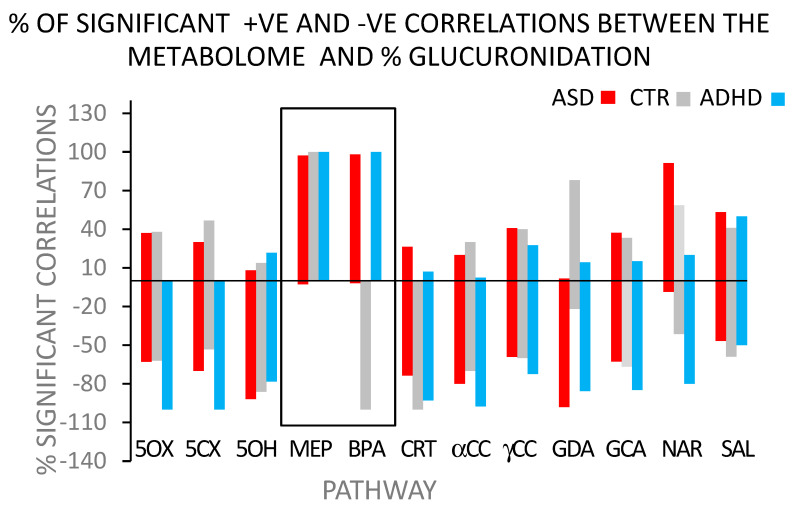
Percent of total number of Spearman’s rank-order correlations from Figure 1, either positive or negative, by glucuronidation pathway for total metabolome. Abbreviations are the same as in Figure 1.

However, the metabolome was not without a structure. The structure became apparent when the distributions of positive and negative correlations were examined separately (Figure 2). The distribution of positive and negative correlations was defined as the percentage of the total number of correlations that were either positive or negative. The sum of the % positive plus % negative correlations equaled 100%.

A significant observation was how different BPA and MEHP were from the other 10 pathways and from each other. They were similar to each other in how they related to glucuronidation efficiency for both ASD (red) and ADHD (blue). In contrast with the other 10 pathways, virtually no negative correlations with ASD (red) and ADHD (blue) for the BPA and MEHP pathways were found. Nearly all the correlations for ASD and ADHD with BPA and MEHP were positive. However, the correlation patterns with BPA and MEHP differed sharply for ASD and ADHD in their relation to the control group (grey). For BPA, the correlations were negative; for MEHP, the correlations were positive. Collectively, the metabolomic analyses showed that BPA (and MEHP) have a complex but well-defined effect on the metabolome.

## 6. Discussion

Multiple factors, one of which is BPA, have been associated with the diseases discussed above. An association does not imply causality, but an association provides a rationale for asking what the underlying mechanism is.

The glucuronidation pathway exhibits heterogeneity at two points: the actual glucuronidation step and the efflux of the glucuronide product from the cell. Both UGT glucuronidation enzymes and the MRP family of efflux transporters are widely distributed in tissues and have multiple functional isoenzymes with many SNPs. Because the substrates for glucuronidation are often toxic, the process needs to be highly efficient. The isoenzymes involved have multiple overlapping substrates. This heterogeneity provides protective redundancy against unexpected toxin excesses. The availability of collateral pathways increases the likelihood of being able to detoxify novel toxins for which there is no genetically predefined pathway.

There are two ways to interpret the findings of associations between BPA glucuronidation efficiency, the metabolome, and disease: a ‘direct’ pathway and an ‘indirect’ pathway.

In the ‘direct’ pathway, free BPA is the actual causative agent. It functions downstream from the glucuronidation step by acting as an endocrine disruptor at some unknown site to cause the disease. In the ‘indirect’ pathway, BPA is not the causative agent. It is a marker for another compound that is the cause and is glucuronidated by the same or very similar set of glucuronidation enzymes as for BPA.

### 6.1. The Direct Pathway

Compromised BPA detoxification is likely to lead to higher concentrations of free BPA in vulnerable tissues, just as it does for drugs. Numerous animal studies have shown BPA to act as an endocrine disruptor [116,119,120,121]. It is, therefore, reasonable to argue that decreased BPA detoxification leads to increased exposure of vulnerable sites in tissues to free BPA, where it can function as an endocrine disruptor. The combination of chronic higher-than-normal levels of free BPA, accompanied by minor distortions downstream from the site of endocrine disruption, creates an environment that favors chronic minor changes in brain metabolism, which, over time, eventually leads to pathology.

### 6.2. The ‘Indirect’ Pathway

In the ‘indirect pathway’, BPA is not the causative agent. BPA serves as a marker for a different configuration of UGTs and/or efflux enzymes from individuals without disease. Compromised BPA glucuronidation is a marker for the glucuronidation efficiency of another unknown compound detoxified by this particular combination of glucuronidation isoenzymes. The compromised detoxification of this unknown endogenous metabolite in the glucuronidation step is responsible for the disease. The available evidence supports the ‘indirect’ pathway.

### 6.3. Evidence Favoring the ‘Indirect’ Pathway

(1) BPA is a relatively inert chemical. Environmental concentrations and exposure are very low, and hence, tissue concentrations are also low. It takes high doses of BPA to produce ‘effects’ in rodents, and no rodent studies have been able to faithfully replicate any of the human diseases listed in Table 1 [43,44,45].
(2) The association of BPA with disease is not unique to BPA. Some of the newer polyphenol analogs of BPA, introduced to replace BPA, are also associated with ADHD [130]. Zebrafish are one of the animal models used to study BPA and phthalates, and parallels in their neurologic effects have been noted. All Zebrafish phthalates and several BPA variants interfere with dopaminergic pathways [131,132]. How many other compounds with similar effects are there? The more compounds there are, the less support there is for the simple direct pathway of elevated levels of BPA somehow interfering with a specific metabolic step to yield a disease.
  The disease differentiating step would probably be either BPA (and MEHP) acting as an endocrine disruptor at different sites (the direct pathway) or differences in the glucuronidation isoenzyme distribution and, hence, a slightly different effect on the metabolism of whatever endogenous compound BPA and MEHP glucuronidation are markers for (the indirect pathway).
(3) The metabolome reflects metabolism within the body. The findings from the previously mentioned studies of ASD (study #3) and ADHD (study #4) on the relationships between toxinogen glucuronidation and the urinary metabolome and ASD and ADHD support the ‘indirect’ pathway. The expected findings from metabolomic studies for the ‘direct’ pathway are either evidence of a simple lesion or an uninterpretable mess. Uninterpretable data is a possibility because of the wealth of data provided by the metabolome, the relatively small number of subjects, and disease heterogeneity. Disease heterogeneity is particularly true for ASD, which is an umbrella term for a variety of closely related diseases. The dataset is complex but not an ‘uninterpretable mess’.
  For the ‘indirect’ pathway, the expected findings show evidence of relationships with BPA at multiple metabolic sites or an uninterpretable mess. The metabolomic results presented above, although complex, are not random, nor are they unique. A metabolomic study on the effects of low levels of BPA on the metabolome of disease-free African-American women also found low-dose BPA exposure to affect multiple metabolic pathways [133]. Findings of widespread effects on the metabolome from low-dose BPA exposure have also been found in rodent studies [134,135,136]. These observations are consistent with BPA having a well-defined, systemic rather than a specific effect on intermediary metabolism.
(4) Support for the concept that BPA glucuronidation detects systemic metabolic disease-related differences comes from a comparison of the correlation distribution patterns found with ASD and ADHD (Figure 2). Finding so many correlations paralleling each other (i.e., >95% correlations in the same direction) is indicative of a systemic effect of BPA on intermediary metabolism.
  Further support for a clear distinction between how BPA and MEHP affect glucuronidation efficiency is that there was no correlation between the glucuronidation efficiencies of BPA and MEHP (r^2^ < 0.02), indicating different systemic effects on the metabolome. There is a clear difference between the pathways of MEHP and BPA glucuronidation. The metabolome has a complex but clearly defined structure.
  The ‘indirect’ pathway explains these differences by attributing them to differences in UGT isoenzyme distribution patterns. More than one pattern can metabolize BPA, but the configurations may differ in their specificity for other substrates. The direct pathway cannot easily explain the systemic effect of low-dose BPA on the metabolome.
(5) This variability in the components of the glucuronidation pathway can account for the observations of multiple diseases correlating with BPA exposure. Just as UGT and transporter distributions can determine the therapeutic effectiveness of drugs metabolized by the glucuronidation pathway [66,67,87], the same process can also lead to endocrine environments favoring disease development (ASD, ADHD, PCOS, PD, and AD), with the actual disease being a function of a particular endocrine environment.
(6) The indirect pathway provides a simple explanation of how a single, relatively inert compound can be associated with several major diseases. Multiple combinations of isoenzymes can be expected to show BPA glucuronidation capability. This variability may not affect overall BPA glucuronidation, but different variants could affect the degradative glucuronidation of various products of intermediary metabolism. The disease potential depends on the product. Plasticity could account for BPA being associated with multiple disparate diseases. The direct pathway does not provide a simple explanation for how BPA could be associated with multiple diseases.

### 6.4. The Role of Steroids

For the diseases listed in Table 1, the actual causative chemical(s) is/are not known. The above arguments favor the agent being a product of intermediary metabolism metabolized by the ‘BPA’ pathway (indirect pathway). The most likely endogenous compound(s) is/are steroid hormone(s). Numerous publications have described how BPA (and MEHP) can act as disruptors of estrogenic and, to a lesser extent, androgenic steroid-mediated processes [116,119,120,121,137]. Steroid hormones are central to multiple processes in all tissues of the body, from gene expression to development and the maintenance of homeostasis. Steroids are hormones, often made at one site and active at another site, which can include the brain. Steroids can easily cross the blood–brain barrier [138].

The particular UGTs and efflux transporters involved in the glucuronidation of BPA and MEHP are those associated with steroid glucuronidation [67,139]. There is extensive literature documenting ASD- and ADHD-related differences in steroid metabolism for ASD [140,141], ADHD, PCOS [105,142], PD [143,144], and AD [145,146]. Indeed, for ASD it has been suggested that differences in the distribution of UGTs and/or efflux transporters could be part of the disease process [141]. If, as we suggest, the diseases listed in Table 1 share a common mechanism, then the key metabolic control point is at the glucuronidation step.

Over a long incubation period, which is the situation for the diseases listed in Table 1, minor differences in the endocrine environment, secondary to differences in the isoenzymes involved in the multi-step glucuronidation process, could lead to chronic small effects on the endocrine environment. Such variations can be beneficial (drug metabolism), neutral, disadvantageous (disease), or, in extreme cases, lethal.

The diseases listed in Table 1 are not lethal. From an evolutionary perspective, they are not even serious. They do not interfere with species’ survival. Rather, they complicate the luxury of the Western lifestyle. The childhood diseases ASD and ADHD do not prevent children from maturing and reproducing. PD and AD occur, for the most part, late in life after the reproductive years are over. PCOS may be an exception, which, in less developed societies, is compensated for by high fertility rates. For the other four diseases considered here, it is largely social interactions that are compromised. The causative biochemical steps are weak, enabling long-term survival in the pre-disease and disease states. 

The concept that neurodevelopmental and neurodegenerative diseases share common etiologies has been suggested by several authors [1,2,3,4,5,6,7,8,9,10]. Indeed, the US NIH-National Institute of Aging recently sponsored a workshop on this very topic.

The support for the concept that the metabolic lesion responsible for the diseases listed in the table is due to isoenzyme differences in UGTs and efflux transporters comes from studies on drug metabolism. The focus of drug studies is to understand the variability in drug effectiveness and toxicity. There is no uncertainty as to the importance of the variance in UGTs and efflux transporters in the metabolism of drugs processed via the glucuronidation pathway. The ‘indirect’ pathway is a variant of this well-characterized pathway.

### 6.5. Limitations

There are several limitations to the above discussion.

(1). Firstly, and most importantly, the above analysis only applies to situations where there is evidence of compromised BPA (and/or MEHP) glucuronidation. The proportion of the total number of patients with each disease fitting into this category is not known, but, as previously pointed out, it must be a significant proportion because evidence for compromised glucuronidation is easily detected in moderate-sized studies [105,107,122,147].
(2). The currently available data are fragmented. Proof of the hypothesis requires the measurement of glucuronidation enzymes’ distribution patterns (UGTs and efflux transporters) plus the glucuronidation efficiency in the same subject. This has not yet been carried out. However, what is available is enough fragmentary information to support a distinctive pattern of events that could lead to disease.
(3). For the two neurodevelopmental and two neurodegenerative diseases, the site of injury is the brain. The fifth disease, PCOS, is not a neurological disease. If the disease-causing lesion is hepatic glucuronidation, either free BPA (the direct hypothesis) or an unknown endogenous compound (the indirect hypothesis) will be in the circulation and will attack vulnerable tissues (e.g., the brain or ovaries). There are many steps between a problematic isoenzyme configuration in the liver and disease in a tissue. The analysis presented here provides no information on those details.
(4). The analysis treated the five diseases as single entities. This is certainly not true for ASD, which is a family of closely related diseases, but it is probably true for AD and PD, where there is a single biochemical product (AD; amyloid) or impacted pathway (PD; dopaminergic).
(5). The evidence supporting an association of AD with BPA is not as strong as with the other four diseases.
(6). The potential role of host disease defense mechanisms in the etiology of the disease has not been considered.

## 7. Conclusions

The title of this paper asks the following question: does chronic exposure to bisphenol A cause human disease? On balance and with some qualifications (see above), the answer is no. Where there is an association with BPA (or MEHP) for the diseases listed in Table 1, the diseases are of genetic and not environmental origin. The cause is not exposure to BPA. BPA is ubiquitous in the modern environment, so exposure is universal. Rather, BPA serves as a marker for a genetically determined isoenzyme distribution pattern that perturbs the metabolism of one or more endogenous metabolites in a way that creates a metabolic environment that favors disease development over an extended time period.

It is a marker because there is an overlap between the enzymes involved in BPA glucuronidation and the glucuronidation of ‘unknown endogenous substrate(s)’ at the level of UGTs and efflux transporters’ isoenzyme distributions. One or more steroids are the most likely endogenous substrates. Numerous animal studies have shown BPA to be a broad-spectrum endocrine disruptor of steroid-mediated processes.

The analysis presented here suggests a road map to test the viability and importance of this conclusion. Glucuronidation efficiency, together with UGT and transport protein distributions in a disease in the same subject, should be measured, and the results should be compared against those of healthy controls.

## Figures and Tables

**Table 1:** Annual % increases in the prevalence of the 5 diseases over the period of ~1995–2022.

Disease	Period	% Increase	Ref.
ASD	2000–2020	15.7	[15]
ADHD	1997–2022	3.4	[16,17]
PCOS	1990–2019	1.8	[21]
PD	2000–2020	7.7	[18]
AD	2000–2018	4.0	[20]

**Table 2:** Human diseases where associations with BPA have been found and potential mechanisms identified in animal studies (adapted from Rebolledo-Solleiro’s study [49]).

**DISEASES**
**Behavioral effects caused by BPA** Memory and learning disorders Anxiety and depressive-like behaviors Socio-sexual behavior
**BPA and neurodegenerative disorders** Parkinson’s disease (PD) Amyotrophic lateral sclerosis (ALS) Multiple sclerosis (MS) Alzheimer’s disease (AD)
**BPA and neurodevelopmental disorders** Autism spectrum disorders (ASDs) Attention-deficit/hyperactivity disorder (ADHD)
**Diseases of the reproductive system** Polycystic ovary syndrome (PCOS)
**POTENTIAL MECHANISMS IDENTIFIED FROM ANIMAL STUDIES**
Endocrine-related mechanisms Epigenetic mechanisms Mitochondrial pathways Calcium and oxidative stress pathways Inflammatory response pathways
